# The effects of nanopillar and nanopit arrays on the morphology and osteogenic differentiation of adipose-derived stem cells

**DOI:** 10.1186/s42649-026-00122-0

**Published:** 2026-01-24

**Authors:** Jihun Kang, Young-Shik Yun, Eun-Hye Kang, Jihye Lee, Deok-Jin Jeon, Seungmuk Ji, Yong-Oock Kim, In-Sik Yun, Jong-Souk Yeo

**Affiliations:** 1 School of Integrated Technology, College of Computing, Yonsei University, Incheon, 21983 Republic of Korea; 2 BK21 Graduate Program in Intelligent Semiconductor Technology, Yonsei University, Seoul, 03722 Republic of Korea; 3 Department of Plastic & Reconstructive Surgery, College of Medicine, Yonsei University, Seoul, 03722 Republic of Korea

**Keywords:** Nanopillar array, Nanopit array, Adipose-derived stem cells, Cell morphology, Osteogenic differentiation

## Abstract

Nanotopographic control of cell behavior offers great potential in designing biomimetic scaffolds for cell therapy. However, the behavior of cells on different nanotopographies is not fully understood. In this study, we investigated the effect of nanostructures on human adipose-derived stem cells (ASCs) by directly comparing nanopillar and nanopit arrays. Morphological changes, cell viability and early osteogenic differentiation of ASCs have been analyzed on the nanostructures. Nanopit arrays were found to increase cell areas and promote early osteogenic differentiation more than nanopillar arrays. Analysis of focal adhesion (FA) formation indicated a larger increase in total area as well as the number of FAs during cell spreading on nanopit arrays. The maturation of FA is related to cellular traction forces, which are known to stimulate osteogenic induction through the RhoA-ROCK pathway. We conclude that ASCs can spread more on the nanopit array than on the nanopillar array due to the presence of continuous adhesive paths on the nanopit array, which is associated with increased expression of RUNX2 as an early osteogenic marker. Our results suggest that a connected path in nanopit arrays plays a critical role in controlling stem cell behavior compared to nanopillar arrays. A comparative understanding of nanostructures can provide a guideline for designing an artificial substrate for osteogenesis and tissue engineering.

Mesenchymal stem cells (MSCs) have the potential to differentiate into desired cell lineages so that they can be used for cell-based therapeutics, including bone engineering (Dalby et al. 2007). For successful tissue regeneration, it is essential to understand stem cells’ interactions with the external environment and to develop biomimetic platforms for regulating cellular responses (Yang et al. 2013; Chen et al. 2014; Qian et al. 2017). Topography and mechanical properties of substrates as biophysical cues of the external environment have been widely investigated (Engler et al. 2006; Hu et al. 2009; Abagnale et al. 2015). According to these studies, biophysical cues can modulate differentiation of stem cells with changes in cell morphology. Recently, a correlation between cell morphology and cellular function was reported, showing that increased cell size (Marklein et al. 2018) and decreased aspect ratio (AR) (Abagnale et al. 2015) in stem cells enhance osteogenic differentiation. The morphological changes are related to the generation of traction force by the formation of focal adhesions (FAs) (Rape et al. 2011). The mechanical force may enhance RhoA-ROCK (Ras homolog gene family member A- Rho kinase) signal pathway for osteogenic differentiation (McBeath et al. 2004; Guvendiren and Burdick 2012).

Over the past years, nanotopography, as one of the biophysical cues, has been employed to investigate the interaction between cells and materials (Chen et al. 2014; Qian et al. 2017). Feature size has been the standard parameter for the analysis of such interactions (Kuo et al. 2014; Abagnale et al. 2015). A disordered nanopit array was reported to promote early osteogenesis of MSCs without osteogenic-inducing factors (Dalby et al. 2007). Anisotropic structures of nanofibers and nanoridges have been shown to produce alignment effects, enhancing desired cellular responses (Zhu et al. 2005; Andalib et al. 2016). However, most of the studies focused on a single type of nanostructure or on variations in pitch, and a direct comparison between nanopillar and nanopit arrays with identical materials and lattice symmetry has rarely been reported. Thus, a more systematic understanding is needed to optimize nanotopographical design to control cellular responses.

In this study, the effects of nanostructures on morphological changes of human adipose-derived stem cells (ASCs) were investigated by comparing inverted nanostructures of nanopillar and nanopit arrays. The nanostructures have the same symmetry in two-dimensional arrays with various pitches. For the formation of FAs, nanopit arrays provide a connected path between adhesive areas, while nanopillar arrays provide discontinuous adhesion sites. We have analyzed cell morphology, cell viability, early osteogenic differentiation, and focal adhesion of ASCs to correlate nanostructure parameters with cellular functions. The correlation identified in our data may provide nanotopographical cues for the design of an artificial substrate to control stem cell osteogenesis.

## Results

### Inverted nanostructures of nanopillar and nanopit arrays

**Fig. 1:**
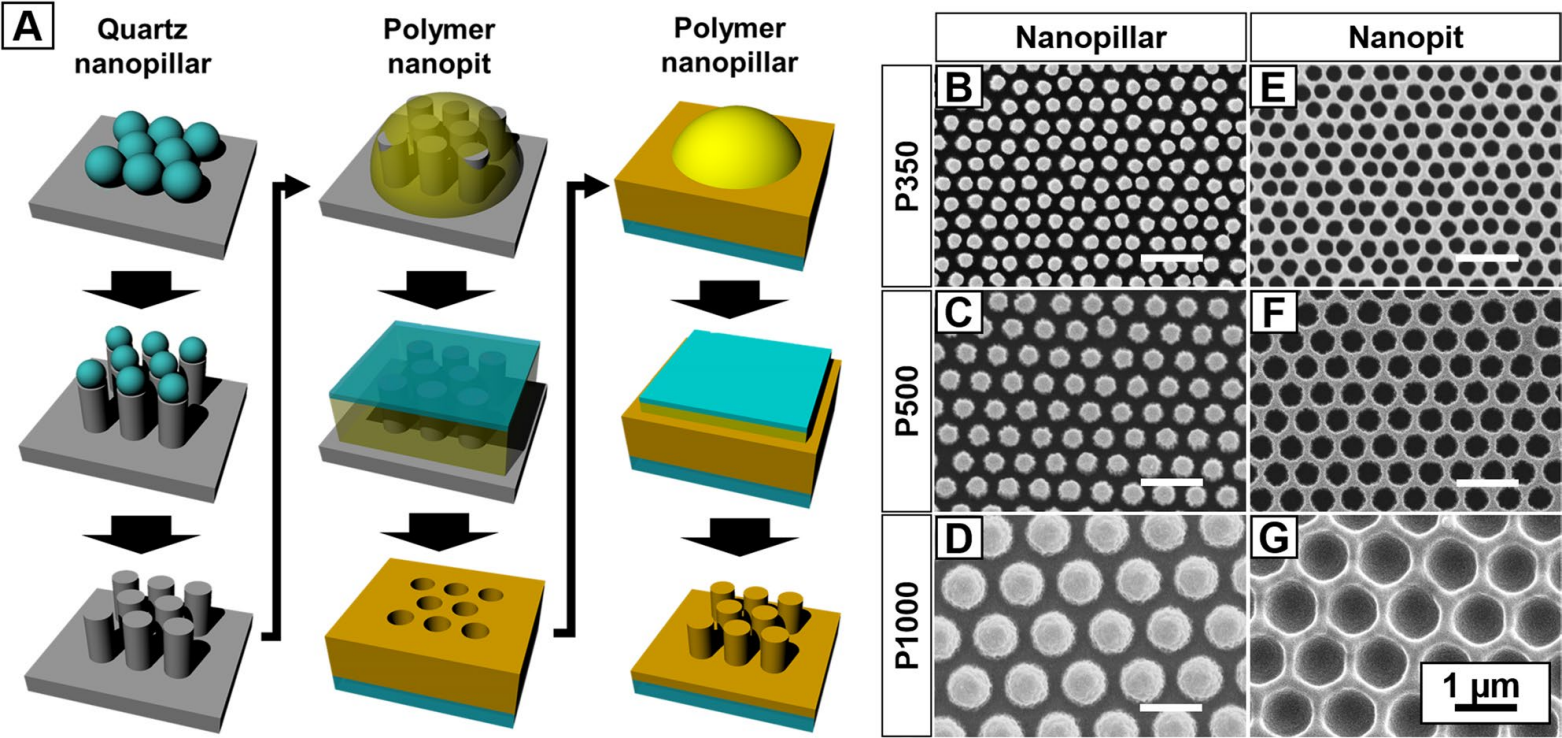
Images of inverted nanostructure substrates of nanopillar and nanopit arrays. **A** Schematic illustration of their fabrication processes. **B**-**G** SEM (scanning electron microscopy) images of nanopillar and nanopit arrays with pitches of 350, 500, and 1000 nm

Figure 1 shows the fabrication process for inverted nanostructures of nanopit and nanopillar arrays. Three quartz master molds were fabricated using polystyrene nanobeads with the diameters of 350, 500, and 1000 nm to control their pitches (Fig. 1A). The resultant mean diameters were 208 ± 9 nm (B), 292 ± 12 nm (C) and 623 ± 23 nm (D) for the polyurethane acrylate (PUA) nanopillar arrays and 253 ± 11 nm (E), 359 ± 12 nm (F) and 787 ± 19 nm (G) for the nanopit arrays, respectively. As the nanopillar arrays were imprinted using the stamp mold of nanopit arrays made from the quartz master mold, the nanopillars had slightly smaller diameters with the nanogaps of 142, 208, and 377 nm between the top surfaces of the respective nanopillar arrays. The inverted nanostructures were fabricated in various pitches using the same material to analyze the effect of topographical differences primarily. The surfaces of all the nanostructures were coated with fibronectin for cell adhesion via integrin binding.

### Nanotopographical effects on the morphological change of ASCs using nanopillar and nanopit arrays

**Fig. 2:**
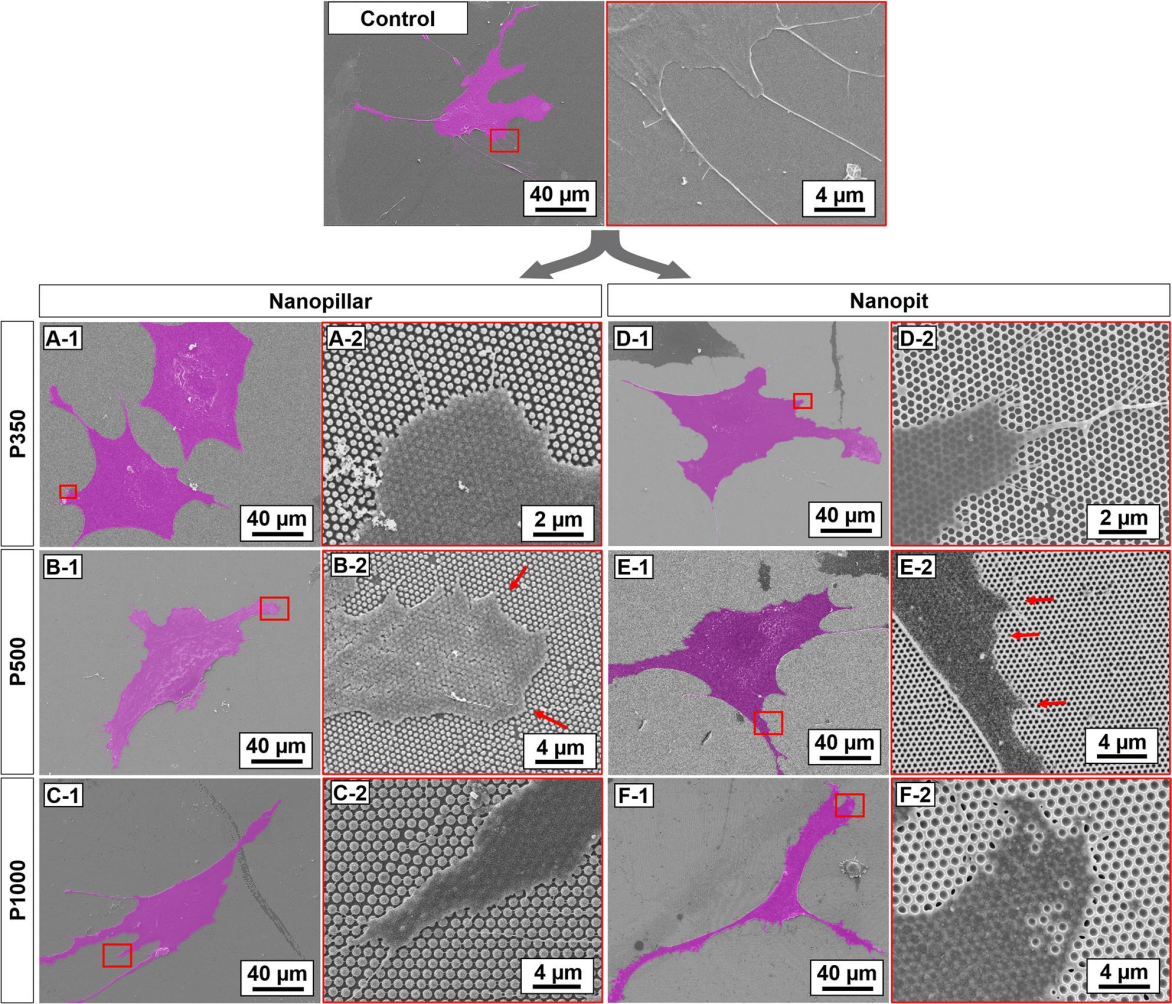
SEM images of ASCs on nanostructures. **A**-**C** ASCs on nanopillar arrays. **D**-**F** ASCs on nanopit arrays; images from A-1 to F-1 on the left side show the morphological changes of ASCs. The magnified SEM images from A-2 to F-2 in red boxes on the right from the nanopillar and nanopit arrays correspond to the red outlined part of the respective images on the left. To clearly show the interaction of the cell and the smaller nanostructure with a 350 nm pitch, the images in A-2 and D-2 are magnified twice as large as B-2, C-2, E-2, and F-2

Figure 2 shows the morphological changes of ASCs for 24 h cultivation on nanostructures. The images on the left side (A-1 to F-1) provide an overview of representative examples showing the morphology of ASCs on different nanostructures. The images on the right side (A-2 to F-2) are magnified images from the red-outlined part of ASCs in the images on the left. Periodic nanostructures provide contact guidance so the ASCs spread following the symmetry of nanostructures in comparison with the flat surface shown on the top as a control.

ASCs spread well on smaller nanostructures with triangular shape at the edges of cell spreading (Fig. 2 A-1, D-1), while cell morphology becomes slender on both nanostructures at 1000 nm pitch (Fig. 2 C-1, F-1). Magnified images provide more details of ASCs interacting with the nanostructures. For the nanopillar arrays, filopodia tend to grow along the symmetry of the nanostructured substrate (Fig. 2 A-2, B-2). For the nanopit arrays, filopodia not only extend along the wall between the nanopits but also grow in a straight line crossing the nanopits when the wall is relatively small compared to the width of extending filopodia (Fig. 2D). As indicated by the red arrows in Fig. 2 B-2 and E-2, the effect of nanotopography on modulating the spreading edge of a cell is clearly visible with the triangular-shaped protrusion of filopodia that follows the underlying symmetry of the nanostructures for a 500 nm pitch. For a smaller pitch of 350 nm, cell spreading may not necessarily follow the underlying nanostructures; the angle at the protruding edge becomes wider on the nanopillar array, or the filopodia span over the holes on the nanopit array, as shown in Fig. 2 A-2 and D-2. For a larger pitch of 1000 nm, the spreading edge of ASCs grows slender with a wider gap between nanopillars or may not cover larger holes in nanopits completely, as shown in Fig. 2 C-2 and F-2.

### ASCs adhesion and osteogenic differentiation on nanopillar and nanopit arrays

**Fig. 3:**
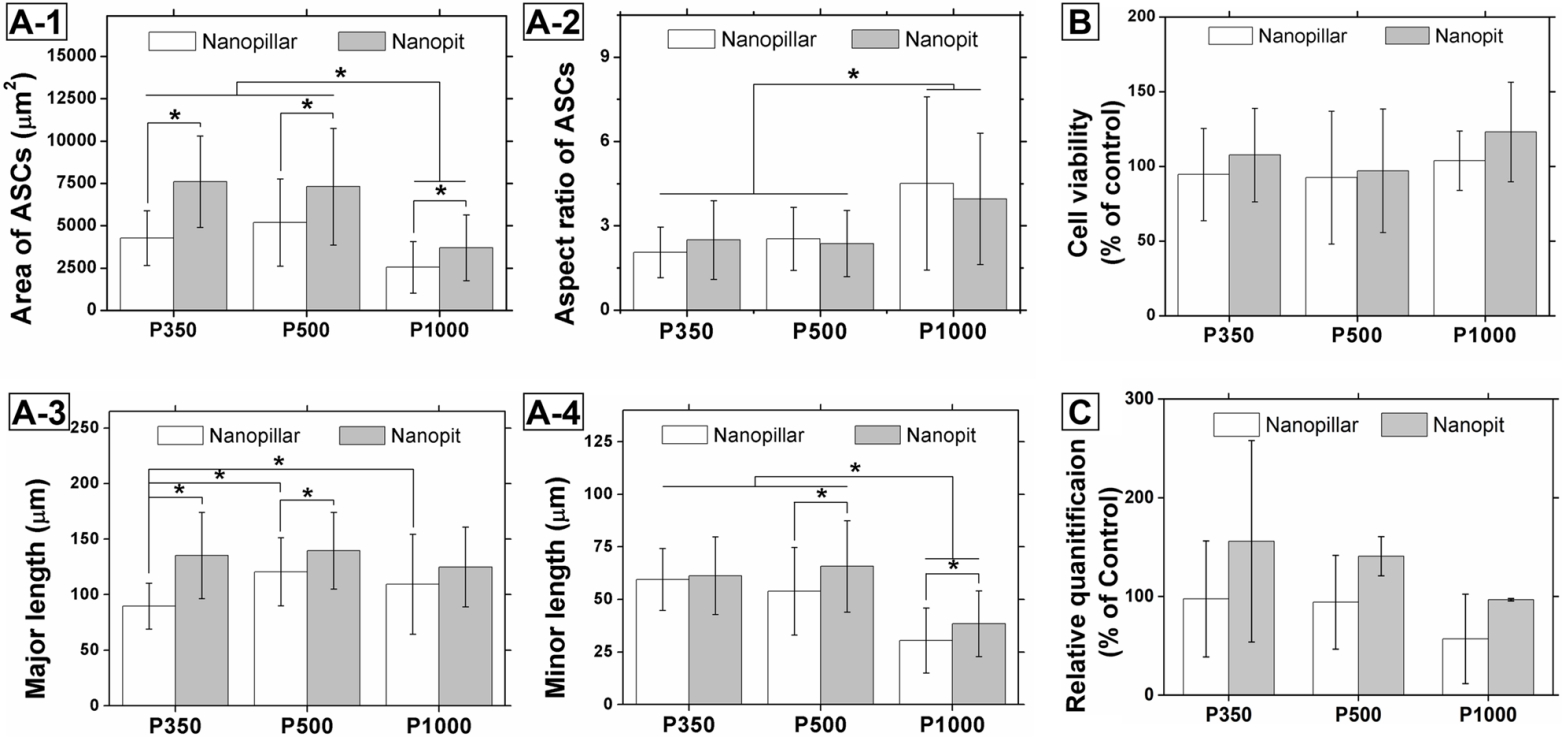
Characterization of adhesion and osteogenic differentiation of ASCs. **A** Morphology of cells defined by cell area, AR, major length, and minor length (*n* = 35 cells per condition, asterisks indicate *P* < 0.05). **B** Cell viability measured through MTT assay (*n* = 6 independent wells per condition). **C** qRT-PCR (quantitative reverse transcription polymerase chain reaction) analysis of RUNX2 expression after 14 days of osteogenic culture to compare osteogenic induction of ASCs on nanopillar and nanopit arrays with various pitches (*n* = 2 independent wells per condition). All error bars represent standard deviations

**Fig. 4:**
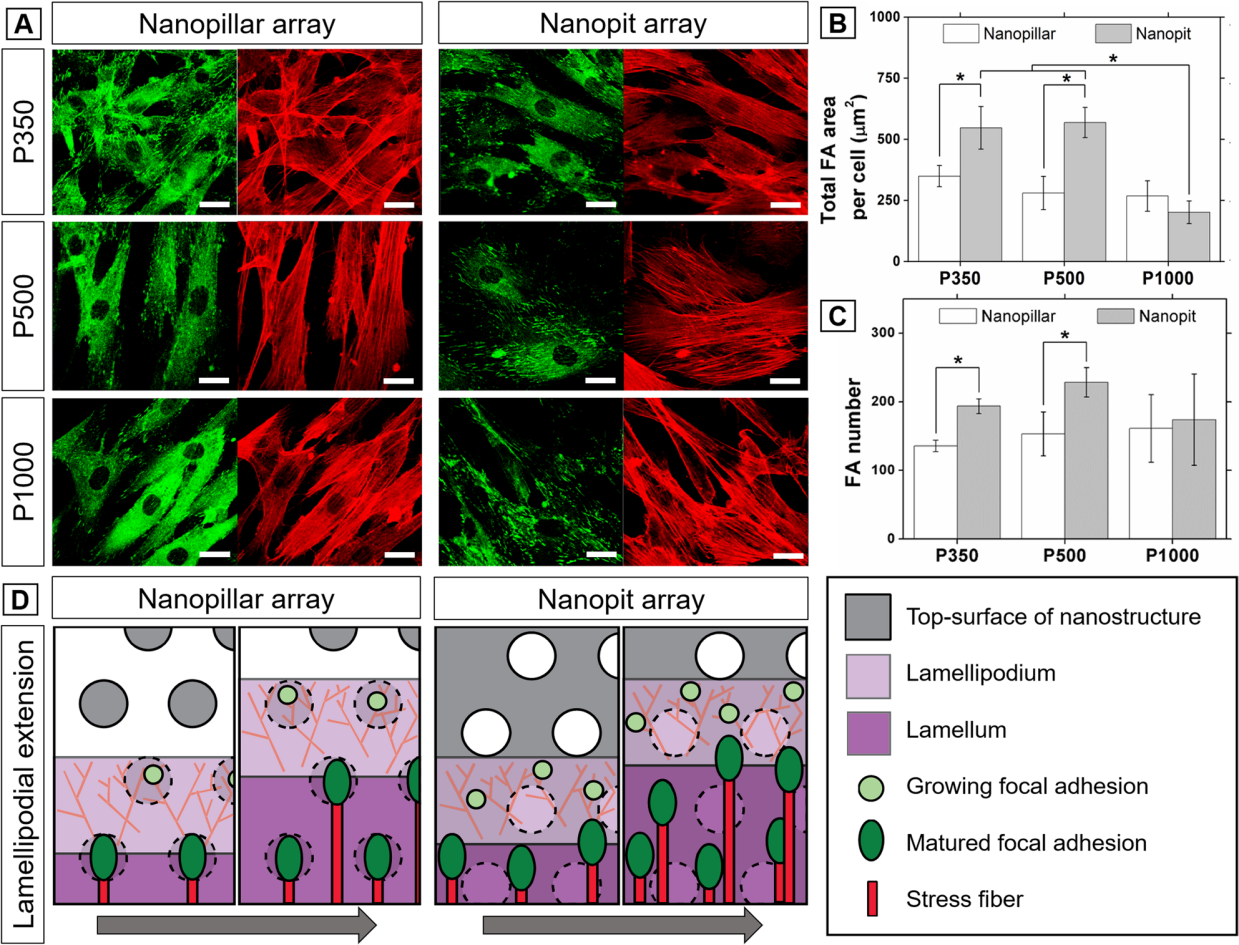
FA analysis and schematic illustration for cell spreading on different nanostructures. **A** Fluorescent images visualized with vinculin (green) and F-actin (red) (Scale bar: 20 μm). **B**-**C** Quantification of FA area per cell and FA number per cell (*n* = 5 cells per condition, with multiple focal adhesions quantified per cell, asterisks indicate *P* < 0.05). Error bars represent standard deviations. **D** Schematic illustration of cell spreading and FA formation, highlighting discontinuous adhesive islands on nanopillars, where adhesion forms on discrete pillar features separated by gaps, versus a connected adhesive path on nanopits, where a continuous ridge network between pits provides a more continuous adhesive interface

Morphological changes in ASCs were quantified by cell area and AR as shown in Fig. 3A. Cell area was measured from SEM images, and AR was calculated as the ratio of the major to minor cell lengths using the ImageJ program, assuming an elliptical shape. Nanopit arrays yielded significantly larger cell areas than nanopillar arrays, regardless of feature size, as shown in Fig. 3A-1. For both nanopillar and nanopit arrays, AR was significantly larger at a pitch of 1000 nm (Fig. 3A-2) due to a larger decrease in minor length at the largest pitch (Figs. 3A and 4). As shown in Fig. 3A, the major length of ASCs shows the most significant difference between nanopillar and nanopit arrays at the smallest pitch of 350 nm, since discontinuous nanopillars led to a larger decrease in rigidity at a smaller pitch than connected nanopits, thus affecting cell spreading (Wong et al. 2014).

Before osteogenic differentiation, the viability of ASCs after 24 h cultivation was evaluated by MTT [3-(4,5-dimethyl-thiazol-2-yl)−2,5-diphenyltetrazolium bromide] assay, as shown in Fig. 3B. Nanotopography did not show a significant effect on cell viability. Osteogenic differentiation was assessed after 14 days of culture in osteogenic medium, as shown in Fig. 3C. The results from area of ASCs in Fig. 3A-1 and the relative quantification in Fig. 3C show a similar trend, suggesting a correlation between cell area and osteogenic differentiation. The expression level of the osteogenesis gene, Runt-related transcription factor 2 (RUNX2), is much higher in nanopit arrays than in nanopillar arrays. This also confirms that the tendency of osteogenesis is most similar to that of cell area among other parameters describing cell morphology. These results are consistent with previous reports on the morphological characteristics of MSCs in response to osteogenic stimulation (Qian et al. 2017; Yang et al. 2019).

### Fluorescent image processing for assessment of FA formation

As previously reported, actin-coupled FAs mediate via the mechanotransduction pathway with traction force for RhoA-ROCK commitments (McBeath et al. 2004; Rape et al. 2011). Figure 4 A shows that FAs and actin stress fibers are formed well in all samples after 24 h incubation. FA formation is characterized by the FA area and number. The total area and the number of FAs are not much different between nanopillar and nanopit arrays with a 1000 nm pitch. However, both the area and the number of FAs increase significantly more on nanopit arrays than on nanopillar arrays at smaller pitches. These results indicate that FAs form more readily on the surfaces of nanopit arrays than on those of nanopillar arrays. Since high surface rigidity has been reported to enable stable cell adhesion and spreading (Wong et al. 2014), connected nanopit arrays appear to provide greater rigidity than discontinuous nanopillar arrays at smaller pitches of 350 and 500 nm. Also, the connected path among nanopit arrays provides adhesion sites for nascent focal adhesions and acts as a pathway for lamellipodial extension, as schematically illustrated in Fig. 4D. The localization of FAs was not directly assessed in this study; however, our previous cross-sectional SEM observations on comparable fibronectin-coated nanostructures suggested that cell–substrate contact was predominantly observed on the top surface with void spaces between features (Yun et al. 2020). Accordingly, recessed regions are unlikely to be the dominant adhesion sites for mature focal adhesions in our system.

## Discussion

Understanding nanostructures and stem cell interactions is key to predicting cellular responses such as cell attachment, proliferation, and differentiation, and to designing biomimetic scaffolds that can facilitate desired responses (Kim et al. 2012; Kang et al. 2020; Yun et al. 2020). In this study, we evaluated the effects of topographical differences between the two representative nanostructures, nanopillar and nanopit arrays, on the morphological changes of ASCs and their osteogenic differentiation. ASCs have been selected to analyze morphological changes and osteogenesis, as they offer many advantages, such as their abundance in the human body, ease of harvest, and potential for differentiation into multiple lineages for biomedical applications (Minteer et al. 2012; Suchanecka et al. 2025). The morphology of a cell is determined by filopodia projection and subsequent cell spreading (Partridge and Marcantonio 2006). Nanopillar and nanopit arrays uniquely offer the localization of periodically arranged adhesion sites for contact guidance, compared to a flat surface as a control. Nanostructures provide contact guidance via integrin binding, promoting filopodia growth. When adhesion sites are available discontinuously, cells need to explore possible attachment sites and find the shortest path between them through filopodial protrusion (Jang et al. 2010; Albuschies and Vogel 2013). Similarly, the leading edges of cells need to find the nearest adhesion sites to form nascent adhesion during lamellipodial protrusion and cell spreading. Therefore, the size of the empty space, such as the gap between nanopillars and the hole within the nanopit array, should determine filopodial and lamellipodial protrusion, and cell spreading.

Figure 2 shows how cells spread on nanopillar and nanopit arrays as their feature size increases. Compared to the better guided spreading of ASCs on nanostructures with a 500 nm pitch (Fig. 2 B-2 and E-2), the spreading edge of ASCs seems to ignore the smaller empty spacing of 142 nm gap for nanopillar array and 250 nm hole for nanopit array, both with the pitch of 350 nm (Fig. 2 A-2 and D-2). On the other hand, the cell spreading was inhibited on nanostructures with 1000 nm pitch, providing relatively larger empty spacing of 377 nm gap for nanopillar array and 787 nm hole for nanopit array, as shown in Fig. 2 C-2 and F-2. These results suggest that the spacing between nanostructures is an important factor in regulating cell spreading. A previous study also reported that cells are not affected by nanotopographical effects for gaps less than 70 nm (Biggs et al. 2010). In our system, nanopit arrays can accommodate larger empty spacing than nanopillar arrays while supporting similar levels of cell spreading, most likely because the connected path of the nanopit arrays provides more continuous adhesion sites even at the same pitch.

The morphology of ASCs was characterized by cell area and AR, as shown in Fig. 3A. The major length was considered to represent cell spreading based on the leading edge of cell (Ridley 2011). The major lengths of ASCs show similar values for nanopit arrays but significantly smaller values for nanopillar arrays at a 350 nm pitch (Fig. 3 A-3). While connected nanopit arrays provide relatively similar mechanical conditions for FA maturation, discontinuous nanopillars become more flexible at 350 nm pitch (Kuo et al. 2014). Such nanopillars with smaller diameter and lower rigidity may inhibit FA maturation and cell spreading at the leading edge, as suggested by previous reports and by our FA analysis (Wong et al. 2014). The minor lengths of ASCs formed by secondary spreading from the leading edge show a significant decrease in nanostructures with a 1000 nm pitch. The combination of major and minor lengths results in a smaller cell area and a higher AR for both nanopillar and nanopit arrays at a 1000 nm pitch, as shown in Fig. 3 A-1 and A-2. The results from the RUNX2 expression for osteoblast differentiation and the area of ASCs show similar trends (Fig. 3 A-1 and C), consistent with the results reported previously (Yao et al. 2013; Yang et al. 2019). In this study, we focused on RUNX2 as an early osteogenic marker since RUNX2 is a well-established transcription factor associated with early osteogenic commitment prior to matrix maturation and mineralization (Meyer et al. 2014). Additional late-stage markers (e.g., alkaline phosphatase, osteocalcin) and mineralization assays will be required in future work to fully establish the long‑term osteogenic outcomes on these nanotopographies. The effect of nanotopography is mediated by mechanotransduction via actin-coupled FAs. Morphological changes of cells reflected through the actin cytoskeleton are known to stimulate osteogenic induction by the RhoA-ROCK pathway (McBeath et al. 2004). However, we did not directly measure RhoA activity or ROCK signaling in this study. Therefore, the involvement of RhoA–ROCK should be interpreted as a literature-supported hypothesis rather than a demonstrated mechanism in our system.

The cell spreading and osteogenic differentiation of ASCs were further studied through FA analysis. Cell areas are significantly higher in nanopit arrays than in nanopillar arrays, regardless of feature size. This difference can be attributed to the connectivity of the adhesive interface. In nanopit arrays, a continuous fibronectin-coated ridge network between neighboring pits provides a connected adhesive path for integrin engagement during protrusion. In nanopillar arrays, adhesion is restricted to isolated pillar tops separated by gaps, which results in discontinuous adhesive paths. As schematically illustrated in Fig. 4D, the connected adhesive path increases the likelihood of forming nascent adhesions ahead of the leading edge and promotes subsequent adhesion growth and maturation during lamellipodial extension. To provide direct three-dimensional evidence for the FA localization depicted in Fig. 4D, future work will employ z-resolved fluorescence imaging and correlative light–electron microscopy. This is supported by the increased FA number on the surface of nanopit arrays (Fig. 4C). The connected paths are believed to provide a similar effect to contact guidance previously reported for the nanoridge array (Yin et al. 2015), since they can be considered as an alignment structure composed of short nanoridges. Figure 4 A shows that actin stress fibers and FAs are sufficiently formed to mediate mechanotransduction in all nanostructures. The mechanical stress applied to these actin cytoskeletons arises from the traction force associated with cell spreading. The traction force tends to increase with increasing formation of FAs (Rape et al. 2011; Trichet et al. 2012). As shown in Fig. 4B and C, the increase in the formation and maturation of FAs on nanopit arrays with 350- and 500-nm pitches can provide greater traction force. This increased traction force on nanopit arrays also affects morphological changes of ASCs as well as their early osteogenic response as shown in Fig. 3 A-1 and C.

## Conclusions

In this work, we compared how two nanotopographies, nanopillar and nanopit arrays, regulate the behavior of ASCs. By correlating quantitative cell morphology, focal adhesion formation, and early osteogenic gene expression, we demonstrated that nanopit arrays support more extensive cell spreading and enhance early osteogenic differentiation than nanopillar arrays. Focal adhesion analysis revealed that the total area and number of adhesions were higher on nanopit arrays at smaller pitches, indicating more efficient maturation of adhesion on surfaces that provide a more continuous adhesive interface. We propose that these connected paths facilitate lamellipodial extension and the formation of focal adhesions, thereby potentially increasing cell traction forces. Such traction‑mediated mechanotransduction is consistent with activation of RhoA–ROCK signaling, a pathway known to promote osteogenic differentiation. Nevertheless, direct assessment of RhoA/ROCK activation will be required to confirm this mechanism in our system. Future studies will directly test this hypothesis by assessing RhoA/ROCK activity and by perturbation experiments using ROCK inhibitors, ideally combined with traction force microscopy. Together, our data support a model in which nanoscale topography governs the fate of ASCs through the coordinated regulation of cell shape, adhesion architecture, and mechanical forces. Future studies that incorporate late‑stage osteogenic markers, matrix mineralization, and in vivo evaluation, as well as systematic variation of stiffness, feature height, and degree of structural disorder, will accelerate the development of clinically relevant osteogenic scaffolds based on controlled nanotopography.

## Methods

### Fabrication of polymer nanostructure arrays

Colloidal lithography has been employed to control center-to-center distance through the size of nanobeads to fabricate quartz nanopillar arrays as master molds (Ji et al. 2012; Lee et al. 2019). Droplets of PUA precursor (MINS-301, Minuta Tech. Co.) were dispensed on the quartz nanopillar mold. A glass coverslip, serving as the substrate, covered the precursor, and the gap between the mold and glass was entirely filled by capillary force (Suh et al. 2009). The imprinted sample was cured under UV exposure (300 mJ/cm^2^) and peeled from the mold carefully. For a nanopillar array, the process was repeated using the PUA nanopit array as a mold with an anti-adhesion coating. The whole fabrication process is schematically illustrated in Fig. 1A.

### Adipose-derived stem cell seeding and cultivation

Human adipose-derived mesenchymal stem cells (ASCs; ATCC^®^ PCS-500-011) were seeded onto nanostructures in the 12-well plate. 2 × 10^4^ cells per well at sixth passage were pipetted onto fibronectin-coated samples. Cell culture was performed in low-glucose Dulbecco’s modified Eagle’s medium (DMEM) basal media in 5% CO_2_ and 37 ℃. The substrates were washed with phosphate-buffered saline (PBS) for 24 h cultivation after seeding and the remaining cells adhered on the substrate were used to assess the next steps. For osteogenic differentiation, DMEM (Thermo Fisher Scientific, USA) was changed to osteogenic differentiation medium after 24 h of cultivation. Then, the cells were incubated for 14 days, with the medium changed at 3–4 day intervals.

### SEM measurement

ASCs were fixed with Karnovsky’s fixative (2% Glutaraldehyde, 2% Paraformaldehyde) for 24 h cultivation. The cells were dehydrated by critical-point drying in a graded series of ethanol (50, 70, 80, 90, and 100%). A thin gold layer was deposited onto the sample by Sputtering in a Cluster system (SNTEK Co., Korea). The morphology of ASCs was observed with SEM (JSM-7100 F, JEOL, Japan).

### Cell viability assay

Cell viability was measured using MTT assay. 500 µL of MTT reagent was dispensed after 24 h cultivation of ASCs. After 3 h cultivation at 5% CO_2_ and 37 ℃, when the purple precipitate was observed, 200 µL of dimethyl sulfoxide (Sigma Aldrich) was carefully dispensed to dissolve formazan salts. The absorbance was measured at 570 nm using a microplate reader (VersaMax™, Molecular Devices, USA).

### Immunofluorescent staining

After 24 h of cultivation, ASCs were fixed with 4% paraformaldehyde in PBS for 20 min at RT and washed three times with PBS. The fixed cells were permeabilized with 0.5% Triton X-100 for 5 min and blocked with 5% bovine serum albumin (BSA) in PBS for 60 min at RT. The cells were then incubated with primary antibody against vinculin (diluted in 5% BSA) and Alexa Fluor 633–conjugated phalloidin (1:150) for 1 h at 37 ℃. After incubation, the cells were washed three times with PBS. The cells were additionally incubated with a fluorescence-labeled secondary antibody (Alexa Fluor 488) diluted in 5% BSA for 30 min at RT in the dark. A mounting solution with DAPI (4′,6-diamidino-2-phenylindole) was used to stain nuclei. The cells were observed through 40x magnified images (numerical aperture 1.2) with a confocal microscope (LSM700, Carl Zeiss, Germany) with ZEN software (Carl Zeiss, Germany).

### Quantitative RT-PCR analysis

Nanotopographical effects on osteogenic differentiation were compared by the result of gene expression using quantitative reverse transcription polymerase chain reaction (qRT-PCR). RUNX2, as a marker of osteogenic differentiation, and glyceraldehyde-3-phosphate dehydrogenase (GAPDH), as a housekeeping gene, were used as qRT-PCR primers (Thermo Fisher Scientific). After 14 days of cultivation in osteogenic differentiation medium, total RNA was isolated from cells using the RNeasy Mini kit (Qiagen, Hilden, Germany) according to the manufacturer’s protocol. 500 ng of RNA was reverse transcribed into complementary DNA using the RT PreMix (Bioneer, Daejeon, Korea), amplified under the conditions of 42 ℃ for 60 min, 94 ℃ for 5 min. qRT-PCR was performed using a Real-time PCR System (Thermo Fisher Scientific, Waltham, MA, USA). Relative gene expression data were analyzed using the comparison Ct (2^−ΔΔCt^) method.

### Image processing

SEM and fluorescent images of ASCs were analyzed using ImageJ (National Institutes of Health, USA). The cells in SEM images with 40× magnification (Resolution: 0.46 pixels per µm, 2782.61 μm × 2226.09 μm) were outlined to measure morphological parameters such as cell area, AR, major length, and minor length. The resolution of the fluorescent images was 3.20 pixels per µm, with dimensions of 160.04 μm × 160.04 μm. FA analysis was performed by quantifying multiple focal adhesions within individual cells from fluorescence images, following the optimized protocol reported by Horzum et al. (Horzum et al. 2014). The resulting focal adhesion area per cell and focal adhesion number per cell were used for statistical comparison.

### Statistical analysis

Results were expressed as mean ± standard deviation and analyzed with one-way ANOVA (analysis of variance) followed by a Tukey post-hoc test for three different sizes of nanostructures and an unpaired t-test for nanopillar and nanopit arrays with the same pitch using OriginPro2017. In all cases, statistical significance was defined as *P* < 0.05.

## Data Availability

The data that support the findings of this study are available from the corresponding author upon reasonable request.

## Abbreviations

ASC: Adipose-derived stem cell
FA: Focal adhesion
MSC: Mesenchymal Stem Cell
AR: Aspect ratio (major length/minor length)
PUA: Polyurethane acrylate
SEM: Scanning electron microscopy
MTT: 3-(4,5-dimethyl-thiazol-2-yl)-2,5-diphenyltetrazolium bromide
qRT-PCR: Quantitative reverse transcription polymerase chain reaction
RUNX2: Runt-related transcription factor 2
RhoA: Ras homolog gene family member A
ROCK: Rho-associated protein kinase
DMEM: Dulbecco’s modified Eagle’s medium
PBS: Phosphate-buffered saline
BSA: Bovine serum albumin
DAPI: 4′,6-diamidino-2-phenylindole
GAPDH: Glyceraldehyde-3-phosphate dehydrogenase
ANOVA: Analysis of variance
